# Real-time fluorometric and end-point colorimetric isothermal assays for detection of equine pathogens *C. psittaci* and equine herpes virus 1: validation, comparison and application at the point of care

**DOI:** 10.1186/s12917-021-02986-8

**Published:** 2021-08-19

**Authors:** Martina Jelocnik, Sharon Nyari, Susan Anstey, Nicole Playford, Tamieka A. Fraser, Keith Mitchell, Anna Blishen, Nina M. Pollak, Joan Carrick, Catherine Chicken, Cheryl Jenkins

**Affiliations:** 1 Genecology Research Centre, University of the Sunshine Coast, Sippy Downs, Qld 4557 Australia; 2 Australia & New Zealand IDEXX Laboratories Pty Ltd, East Brisbane, Qld 4169 Australia; 3 Scone Equine Group, Scone, NSW 2337 Australia; 4 Equine Specialist Consulting, Scone, NSW 2337 Australia; 5 NSW Department of Primary Industries, Elizabeth Macarthur Agricultural Institute, Menangle, NSW 2568 Australia

**Keywords:** Isothermal amplification, Colorimetric detection, Equines, *Chlamydia*, EHV-1, Point of care application

## Abstract

**Background:**

*C. psittaci* has recently emerged as an equine abortigenic pathogen causing significant losses to the Australian Thoroughbred industry, while Equine herpesvirus-1 (EHV-1) is a well-recognized abortigenic agent. Diagnosis of these agents is based on molecular assays in diagnostic laboratories.

In this study, we validated *C. psittaci* and newly developed EHV-1 Loop Mediated Isothermal Amplification (LAMP) assays performed in a real-time fluorometer (rtLAMP) against the reference diagnostic assays. We also evaluated isothermal amplification using commercially available colorimetric mix (cLAMP), and SYBR Green DNA binding dye (sgLAMP) for “naked eye” end-point detection when testing ‘real-world’ clinical samples. Finally, we applied the *C. psittaci* LAMP assays in two pilot Point-of-Care (POC) studies in an equine hospital.

**Results:**

The analytical sensitivity of *C. psittaci* and EHV-1 rt-, and colorimetric LAMPs was determined as one and 10 genome equivalents per reaction, respectively. Compared to reference diagnostic qPCR assays, the *C. psittaci* rtLAMP showed sensitivity of 100%, specificity of 97.5, and 98.86% agreement, while EHV-1 rtLAMP showed 86.96% sensitivity, 100% specificity, and 91.43% agreement.

When testing rapidly processed clinical samples, all three *C. psittaci* rt-, c-, sg-LAMP assays were highly congruent with each other, with Kappa values of 0. 906 for sgLAMP and 0. 821 for cLAMP when compared to rtLAMP. EHV-1 testing also revealed high congruence between the assays, with Kappa values of 0.784 for cLAMP and 0.638 for sgLAMP when compared to rtLAMP. The congruence between LAMP assays and the *C. psittaci* or EHV-1 qPCR assays was high, with agreements ranging from 94.12 to 100% for *C. psittaci*, and 88.24 to 94.12% for EHV-1, respectively.

At the POC, the *C. psittaci* rt- and c-LAMP assays using rapidly processed swabs were performed by technicians with no prior molecular experience, and the overall congruence between the POC *C. psittaci* LAMPs and the qPCR assays ranged between 90.91–100%.

**Conclusions:**

This study describes reliable POC options for the detection of the equine pathogens: *C. psittaci* and EHV-1. Testing ‘real-world’ samples in equine clinical setting, represents a proof-of-concept that POC isothermal diagnostics can be applied to rapid disease screening in the equine industry.

**Supplementary Information:**

The online version contains supplementary material available at 10.1186/s12917-021-02986-8.

## Background

As for other mammalian hosts, infectious diseases are important causes of morbidity and mortality in horses, incurring significant economic losses for stakeholders. Endemic and emerging equine pathogens pose significant risks not only to equine health, but in some cases to humans [1, 2]. Horses are a key part of One Health due to their role in transmission of both viral and bacterial diseases from wildlife to humans [1, 2]. *C. psittaci* (*C. psittaci*) has recently emerged as an equine abortigenic pathogen causing significant losses to the Australian Thoroughbred industry [3–5]. Furthermore, this zoonotic pathogen has attracted attention due to the apparent transmission of *C. psittaci* from equine placental membranes to humans causing severe respiratory illness [6, 7]. Equine herpesvirus-1 (EHV-1) also causes equine abortion as well as morbidity and mortalities in neonatal foals. Despite being highly contagious in horses, and in contrast to *C. psittaci*, EHV-1 does not pose a risk to humans [1, 8]. Nonetheless, EHV-1 is endemic to Australia and is one of the most economically important and prevalent pathogens of horses, posing a major global threat to the equine (Thoroughbred) industry worldwide [8–10].

Methods for nucleic acid detection, such as quantitative real-time PCR (qPCR), remain a ‘gold-standard’ in veterinary diagnostics; however, this testing is usually performed in well-equipped veterinary diagnostic laboratories with molecular capabilities and trained personnel [1, 11–13]. The requirement for specialized equipment and skilled technicians results in significant testing costs and turnaround times before diagnostic results are available, delaying efforts to manage and treat infected animals [12, 14]. Rapid point-of-care (POC) diagnostic tests, performed on simple equipment, allow minimally trained field veterinarians to accurately achieve real-time, lab-quality diagnostic results within minutes rather than hours. As such, the development of field amenable POC tests provides an attractive solution to fill an unmet need in the equine industry [12]. The availability of such POC tests would help with fast and accurate diagnosis of equine pathogens in the field, greatly improve biosecurity and offer cost-effective surveillance [9, 12, 14–16]. Rapid diagnosis is also of importance for veterinarians and others at risk of exposure to zoonotic pathogens in field or farm settings [1, 12, 14].

To date, numerous isothermal amplification and end-point detection methods have been established [11, 13]. Perhaps, it is the robustness, rapidity, high specificity, and simplicity of loop-mediated isothermal amplification (LAMP) that made this versatile technique a rapid diagnostic of choice for a range of human and veterinary pathogens [11, 13, 17, 18]. Briefly, LAMP is a nucleic acid amplification that runs for 30–60 min at constant temperature, where a set of four (or six) different primers binds to six (or eight) different regions on the target gene making target identification highly specific [18]. LAMP assays can be run on (a) real-time fluorometers which provide an option to include a high-resolution melt curve at the end of each amplification run offering additional confirmation of a positive result, or (b) heating blocks which give a colorimetric and/or turbidimetric end-point result for visual interpretation by naked eye [11, 13, 18]. Species-specific isothermal assays were previously proposed for *C. psittaci* [14] and EHV-1 [16, 19, 20], however, these assays are not commonly employed in the equine industry, as comparative studies with ‘real world’ clinical samples have been limited to date [9, 14, 20]. Another reason for the limited POC use of these assays could be the costs associated with the initial setup and/or equipment (e.g. estimated price of a real time fluorometer is USD $12000).

The aim of this study was to compare the previously described *C. psittaci* [14] and newly developed EHV-1 LAMP assays to the reference diagnostic assays. We also evaluate three methods for isothermal amplification for these pathogens using: a) real-time fluorometer; b) commercially available colorimetric mix; and c) DNA binding dye (e.g. SYBR Green) for “naked eye” end-point detection when testing ‘real-world’ clinical samples. Finally, we apply these methods in a pilot POC testing study in an equine hospital diagnostic laboratory.

## Results

### Analytical specificity, limit of detection and stability of the reagents

For *C. psittaci*, we employed the primer set from our previously established LAMP assay for all testing [14], whereas for EHV-1 we designed and compared this new primer set to previously published primers [19]. Our newly designed EHV-1 primers were also predicted to target a very similar gene region as the previous set (Additional files 1 and 2).

**Fig. 1:**
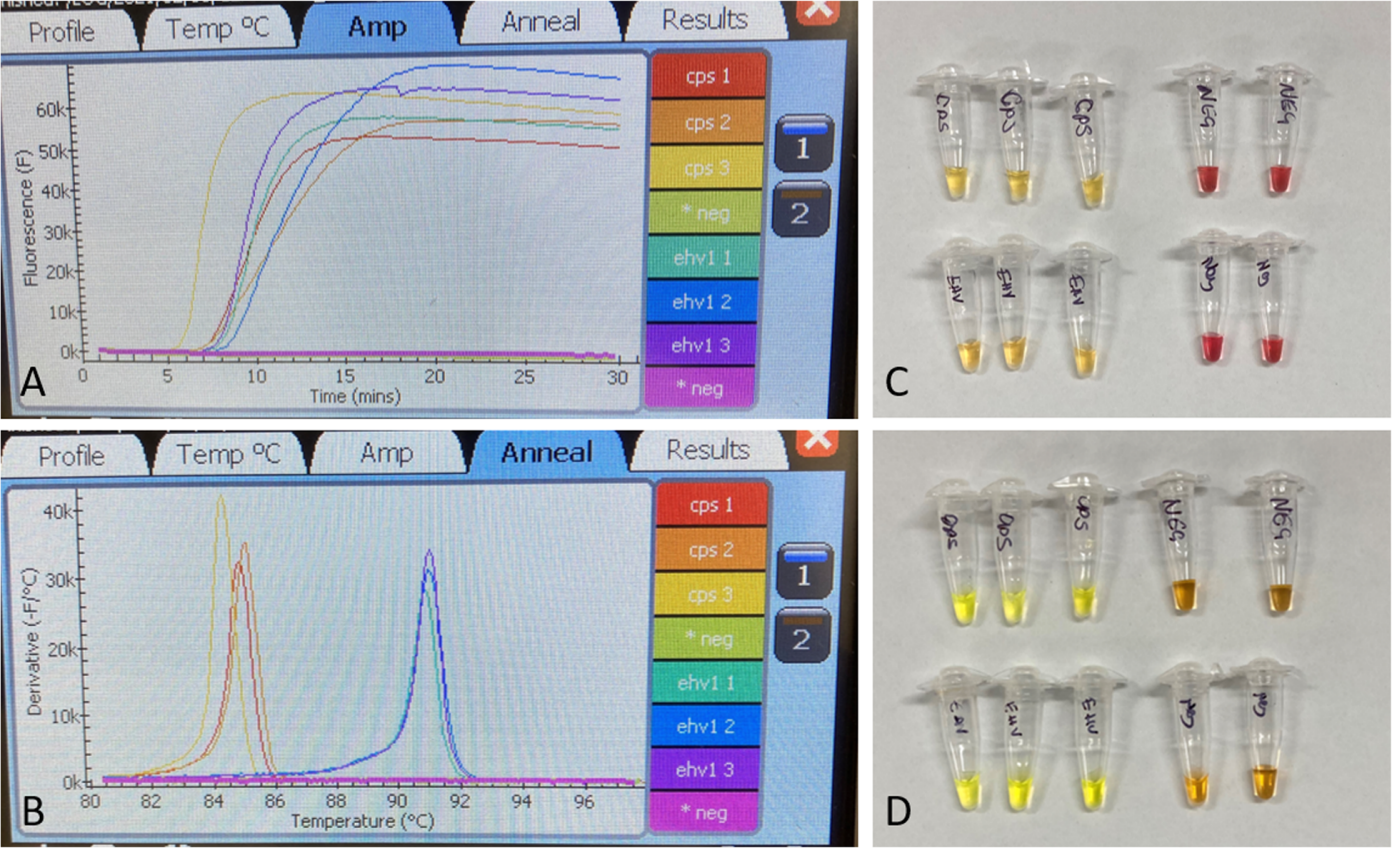
Real-time and colorimetric C. psittaci and EHV-1 LAMP assays. The real-time fluorometer Genie III displays of (A) a *C. psittaci* and EHV-1 amplification curves within 30 min amplification time, and (B) High-resolution melts (HRM). Positive rtLAMP result is denoted with time to amplify (mm:ss) and HRM (°C), whilst negative result does not record either. End-point detection using *C. psittaci* (top row) and EHV-1 (bottom row) (C) cLAMP and (D) sgLAMP assays. cLAMP reaction resulting in yellow colour after amplification denote a positive result, and while pink colour represents a negative test result. sgLAMP reaction showing a bright yellow-green colour denote a positive test result and orange colour depict a negative result. The images were taken with iPhone SE

The detection limits of the three LAMP assays (real-time [rtLAMP]; colorimetric [cLAMP] and SybrGreen LAMP [sgLAMP]) were evaluated using ten-fold serial-diluted, purified and quantified *C. psittaci* or EHV-1 gDNA as template (Fig. 1). Each serial dilution set (10^2^ to 10^− 1^ copies/μl) was prepared separately, with each replicate tested in a separate run.

First, we evaluated detection limit for the real-time LAMP (rtLAMP). The detection limit for both *C. psittaci* and EHV-1 rtLAMP assays (Fig. 1A and B) was equivalent to 1 genome copy/μl template (5/5 replicates, 100% detection), whereas for previously published EHV-1 LAMP assay [19], the detection limit was 10 genome copies/μl template (5/5 replicates, 100% detection) (Additional file 4).

Next, we evaluated the detection limit for the colorimetric LAMP (cLAMP) assays using the thermal block for amplification. The analytical sensitivity of both *C. psittaci* and EHV-1 cLAMP assays (Fig. 1C) was only slightly lower than that of the LAMP assays performed using the Genie III real-time fluorometer with an equivalent to 10 genome copies/μl template (5/5 replicates, 100% detection) representing the limit of detection in each case (Additional file 4). Interestingly, colorimetric amplification using the EHV-1 primer set of Nemoto et al. [19] was unsuccessful with negative controls yielding a yellow colour change after incubation indicating a false positive (pink colour equals a negative test). After evaluating detection limits of the rt-, and cLAMP assays and deciding on the most optimum primer set for further analyses (i.e. EHV-1 primers designed in this study), we also evaluated the detection limit for the LAMP assays using SYBR Green detection (sgLAMP) (Fig. 1D). As for the sgLAMP assays, the detection limit for both the *C. psittaci* and EHV-1 sgLAMP was equivalent to 10 genome copy/μl template (5/5 replicates, 100% detection) (Additional file 4).

To confirm the target specificity of *C. psittaci* primer set and EHV-1 primer set designed in this study, we tested DNA extracted from a range of bacterial and viral species using rtLAMP assays (Additional file 3). We have successfully amplified targeted DNA from the reference strains, tested individually and/or as a mixture with other non-targeted DNA, and no amplification was recorded for any other bacterial or viral species tested (Additional file 4). Finally, we used the *C. psittaci* LAMP assay to test the stability of all three sets of LAMP reagents under refrigeration conditions (4^o^ C) over a six-week period (Additional files 3 and 5). A range of quantified *C. psittaci* DNA samples was tested weekly up to six weeks in order to unambiguously achieve a distinct positive result (*C. psittaci* DNA with higher genome copy number), and more accurately evaluate reagent stability (*C. psittaci* DNA with lower genome copy number). Amplification was continuously achieved weekly for all samples up to the endpoint of six weeks independent of LAMP assay types (Additional file 5) confirming reagent stability for six weeks.

### Diagnostic validation

The reference qPCR diagnostic assays performed in National Association of Testing Authorities, Australia (NATA) accredited Veterinary Diagnostic laboratories were compared to the *C. psittaci* and EHV-1 rtLAMP assays. For *C. psittaci*, a total of 88 DNA samples, extracted from tissue swabs from abortion material from equine abortion cases, were tested by both rtLAMP and the EMAI Veterinary diagnostic laboratory reference diagnostic qPCR assay. Compared to the reference assay, the *C. psittaci* rtLAMP assay showed sensitivity (accuracy) of 100% and specificity of 97.5%, with overall agreement of 98.86% (Table 1). A discrepant result was observed for only one sample, and it was further noted that the qPCR negative result was deemed as indeterminate (Additional file 7).

**Table 1:** *C. psittaci* and EHV-1 LAMP assays in comparison to reference diagnostic qPCR assays

**Reference*C. psittaci*qPCR**				
***C. psittaci*** **rtLAMP**		Positive	Negative	Total
	Positive	48	1	49
	Negative	0	39	39
	Total	48	40	88
	Sensitivity ^a^	100% (92.62–100%)		
	Specificity ^a^	97.5% (86.84–99.94%)		
	Likelihood ratio + ve	40 (95% CI 5.77 to 277.06)		
	Likelihood ratio -ve	0		
	Overall agreement ^b^	98.86% (98.97% PA; 98.73% NA)		
**EMAI Reference EHV-1 qPCR**				
**EHV-1 rtLAMP**		Positive	Negative	Total
	Positive	20	0	20
	Negative	3	12	15
	Total	23	12	35
	Sensitivity ^a^	86.96% (66.41–97.22%)		
	Specificity ^a^	100% (73.54–100%)		
	Likelihood ratio + ve	N/A		
	Likelihood ratio -ve	0.130 (95% CI 0.05 to 0.37)		
	Overall agreement ^b^	91.43% (93.02% PA; 88.89% NA)		
**IDEXX Reference EHV-1 qPCR**				
**EHV-1 rtLAMP**		Positive	Negative	Total
	Positive	3	0	3
	Negative	0	5	5
	Total	3	5	8
	Sensitivity ^a^	100% (29.24–100%)		
	Specificity ^a^	100% (47.82–100%)		
	Likelihood ratio + ve	N/A		
	Likelihood ratio -ve	0.00		
	Overall agreement ^b^	100%		

^a^: sensitivity and specificity are presented with specified Clopper-Pearson (exact) lower and upper 95% confidence limits, ^b^: positive agreement (PA) and negative agreement (NA) are outlined in brackets. N/A: not applicable

For EHV-1, 35 DNA samples, also extracted from swabs from equine abortion cases, were tested by both rtLAMP and the EMAI Veterinary diagnostic laboratory reference qPCR assays, whereas another eight DNA samples were tested by both rtLAMP and the IDEXX Veterinary diagnostic laboratory reference qPCR assays (Table 1, Additional file 7). Compared to the EMAI reference qPCR assay, the EHV-1 rtLAMP assay showed sensitivity (accuracy) of 86.96% and specificity of 100%, with overall agreement of 91.43% (Table 1). Discrepant results were noted for three samples, negative by LAMP but positive by qPCR. With only eight DNA samples, extracted from tissue swabs from mares’ urogenital sites and abortion material, available for testing, the EHV-1 rtLAMP assay showed sensitivity and specificity of 100% compared to the IDEXX reference qPCR assay (Table 1). Two clinical samples (H1 and H2, Additional file 7) also had diagnosed co-infection with EHV-4 and EHV-2, respectively.

For all discrepant samples (one for *C. psittaci* and three for EHV-1), rtLAMP testing was repeated to confirm results, whereas all results from the diagnostic laboratories testing were deemed valid and from conforming runs.

### *C. psittaci* and EHV-1 detection in rapidly processed clinical samples using LAMP assays

**Fig. 2:**
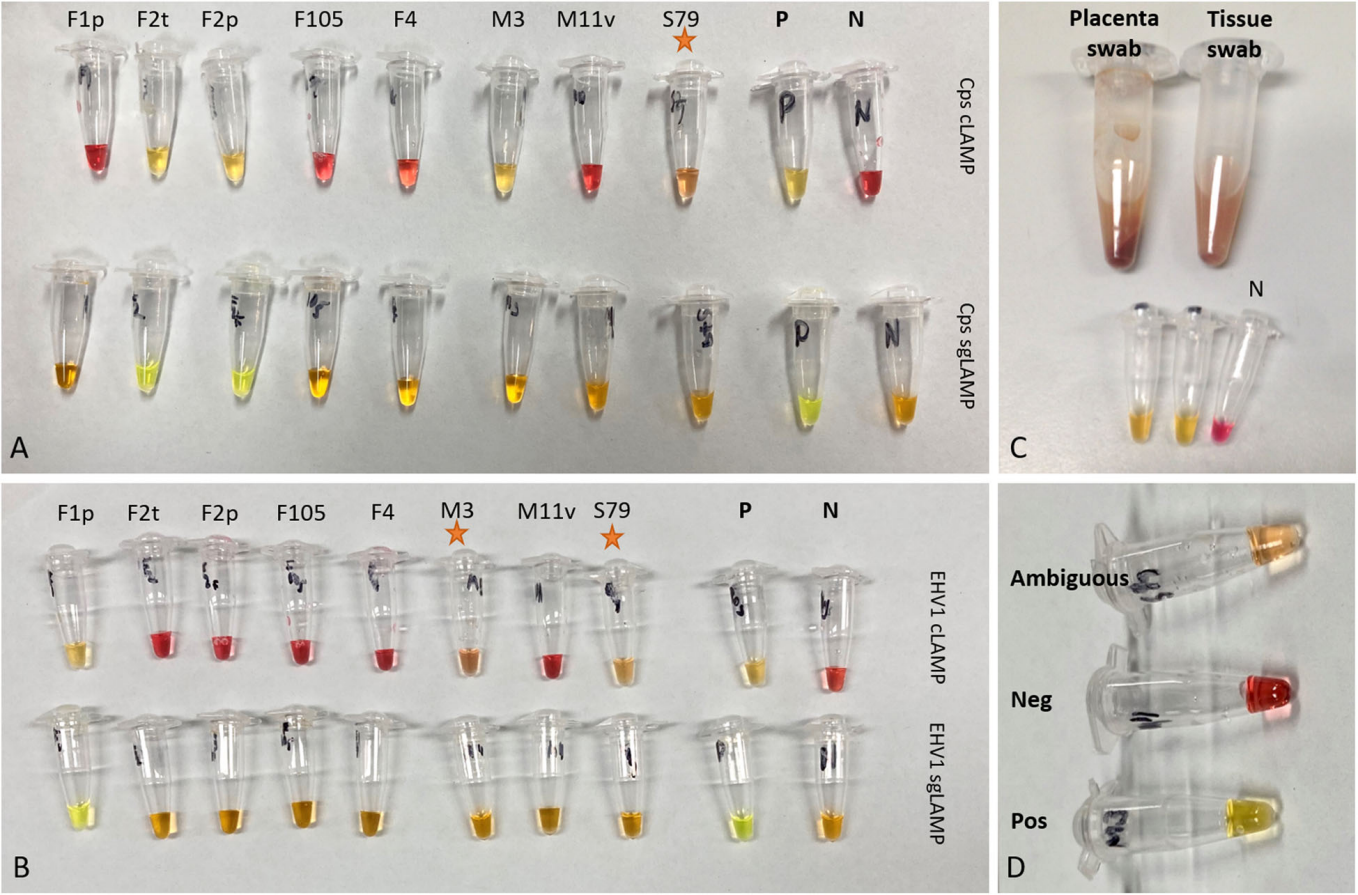
Colorimetric and SYBR Green LAMP assays testing of the same clinical swab suspension samples. cLAMP assay (top row) and sgLAMP assay (bottom row) testing for presence of *C. psittaci* (A), and EHV-1 (B). In (C) *C. psittaci* results of rapidly processed placental and tissue swabs suspensions using cLAMP, and (D) test results of *C. psittaci* cLAMP yielding ambiguous result (orange colour, top), unambiguous negative (pink colour, middle) and unambiguous positive (yellow colour, bottom). Tested swab samples IDs are (from left): F1 tissues, F2 tissues1 and placenta1, F105 placenta, F4 nasal, M3 cervix, M11 vagina, and S79 penile (Table S5). Positive and negative tests ate denoted with P and N, respectively. Sample M3 in EHV-1 cLAMP, and sample S79 in both *C. psittaci* and EHV-1 cLAMPs yielded ambiguous test results (orange colour, indicated by orange stars). The images were taken with iPhone SE

Two penile samples were excluded from cLAMP testing as immediate discoloration of the mix (to yellow and/or orange) was observed upon addition of the swab suspension indicating that template from these types of samples were of low pH. However, samples yielding an orange colour post amplification were deemed “ambiguous” and reported as negative (see samples indicated by orange star in Fig. 2).

### *C. psittaci*

Overall, all three *C. psittaci* LAMP assays testing rapidly processed swab samples were highly congruent with each other (Additional file 8), with Kappa values of 0. 906 for sgLAMP and 0. 821 for cLAMP when compared to rtLAMP as reference test, indicating almost perfect and substantial agreement between the methods, respectively (Table 2). In addition, there was a substantial agreement between sg- and cLAMP as indicated by a Kappa of 0.744.

**Table 2:** Comparison of *C. psittaci* LAMP assays using rapidly processed swab samples

***C. psittaci*cLAMP**^#^	***C. psittaci*rtLAMP**			
		Positive	Negative	Total
	Positive	6	2	8
	Negative	0	26	26
	Total	6	28	34
	Kappa (0.95 CI; p(Kappa))	0.821 (0.584–1.06; 0.00)		
	McNemar’s Chi square (p(Chi sq))	0.500 (0.479)		
	Overall agreement ^b^	94.12% (85.71% PA; 96.30% NA)		
***C. psittaci*sgLAMP**	***C. psittaci*rtLAMP**			
		Positive	Negative	Total
	Positive	6	1	7
	Negative	0	29	29
	Total	6	30	36
	Kappa (0.95 CI; p(Kappa))	0.906 (0.726–1.09; 0.00)		
	McNemar’s Chi square (p(Chi sq))	0.000 (1.00)		
	Overall agreement ^b^	97.22% (92.31% PA; 98.31% NA)		
***C. psittaci*sgLAMP**	***C. psittaci*cLAMP**^#^			
		Positive	Negative	Total
	Positive	6	1	7
	Negative	2	25	27
	Total	8	26	34
	Kappa (0.95 CI; p(Kappa))	0.744 (0.471–1.017; 0.00)		
	McNemar’s Chi square (p(Chi sq))	0.000 (1.00)		
	Overall agreement ^b^	91.18% (80.00% PA; 94.34% NA)		

^#:^ two samples were excluded from analyses as immediate discoloration of the mix (to yellow and/or orange) was observed upon addition the swab suspension; ^b^: positive agreement (PA) and negative agreement (NA) are outlined in brackets

Next, we evaluated the performance of the LAMP assays in comparison to the reference *C. psittaci*-specific qPCR assay. The overall congruence between the three LAMP assays and the *C. psittaci* qPCR assay was high, with agreements ranging from 94.12 to 100% (Table 3). Discrepant results were noted for two samples (F3 Placenta1 and M3 cervix) negative in all assays except cLAMP (Fig. 2), and another sample (M94 clitoris) which was negative in all assays except sgLAMP. The sample, M100vagina, was considered negative despite showing late amplification in rtLAMP at 27 min as this was below 100% detection limit (Additional file 8).

**Table 3:** Comparison of *C. psittaci* LAMP assays using rapidly processed samples to qPCR testing

***C. psittaci*rtLAMP**	***C. psittaci*qPCR**			
		Positive	Negative	Total
	Positive	6	0	6
	Negative	0	30	30
	Total	6	30	36
	Kappa (0.95 CI; p(Kappa))	1 (1; 0.00)		
	McNemar’s Chi square (p(Chi sq))	Inf (0.000)		
	Overall agreement ^b^	100%		
***C. psittaci*cLAMP**^***#***^	***C. psittaci*qPCR**			
		Positive	Negative	Total
	Positive	6	2	8
	Negative	0	26	28
	Total	6	28	34
	Kappa (0.95 CI; p(Kappa))	0.821 (0.584–1.058; 0.00)		
	McNemar’s Chi square (p(Chi sq))	0.500 (0.479)		
	Overall agreement ^b^	94.12% (85.71% PA; 96.30% NA)		
***C. psittaci*sgLAMP**	***C. psittaci*qPCR**			
		Positive	Negative	Total
	Positive	6	1	7
	Negative	0	29	29
	Total	6	30	36
	Kappa (0.95 CI; p(Kappa))	0.906 (0.726–1.087; 0.00)		
	McNemar’s Chi square (p(Chi sq))	0.00 (1.00)		
	Overall agreement ^b^	97.22% (92.31% PA; 98.31% NA)		

^#:^ two samples were excluded from analyses as immediate discoloration of the mix (to yellow and/or orange) was observed upon addition the swab suspension; ^b^: positive agreement (PA) and negative agreement (NA) are outlined in brackets

### EHV-1

EHV-1 testing of rapidly processed clinical swabs revealed high congruence between the three LAMP assay methods (Additional file 8), with Kappa values of 0.784 for cLAMP and 0.638 for sgLAMP respectively when compared to rtLAMP (Table 4). The agreement between sgLAMP and cLAMP assay was moderate, as indicated by a Kappa of 0.520.

**Table 4:** Comparison of EHV-1 LAMP assays using rapidly processed swab samples

**EHV-1 cLAMP**^**#**^	**EHV-1 rtLAMP**			
		Positive	Negative	Total
	Positive	2	1	3
	Negative	0	29	29
	Total	2	30	32
	Kappa (0.95 CI; p(Kappa))	0.784 (0.377–1.191; 0.00)		
	McNemar’s Chi square (p(Chi sq))	0.00 (1.00)		
	Overall agreement ^b^	96.88% (80.00% PA; 98.31% NA)		
**EHV-1 sgLAMP**	**EHV-1 rtLAMP**			
		Positive	Negative	Total
	Positive	2	2	4
	Negative	0	30	30
	Total	2	32	34
	Kappa (0.95 CI; p(Kappa))	0.638 (0.185–1.09; 0.000)		
	McNemar’s Chi square (p(Chi sq))	0.500 (0.479)		
	Overall agreement ^b^	94.12% (66.67% PA; 96.77% NA)		
**EHV-1 sgLAMP**	**EHV-1 cLAMP**^**#**^			
		Positive	Negative	Total
	Positive	2	2	4
	Negative	1	27	28
	Total	3	29	32
	Kappa (0.95 CI; p(Kappa))	0.520 (0.047–0.993; 0.0014)		
	McNemar’s Chi square (p(Chi sq))	0.00 (1.00)		
	Overall agreement ^b^	90.62% (57.14% PA; 94.74% NA)		

^#:^ two samples were excluded from analyses as immediate discoloration of the mix (to yellow and/or orange) was observed upon addition the swab suspension; ^b^: positive agreement (PA) and negative agreement (NA) are outlined in brackets

Compared to EHV-1 qPCR assay, rtLAMP had the highest overall agreement of 94.12% and a Kappa of 0.638; followed by cLAMP and sgLAMP with 90.62 and 88.24% agreements (Kappa of 0.520 and 0.433), respectively (Table 5). Discrepant results were noted for two samples that were negative by all assays except qPCR. Of those, one (F18 placenta) had recorded amplification time and melt in rtLAMP, however this was below the limit of detection and was therefore considered negative. One discrepant sample (F107 placenta) was positive by cLAMP but negative in all other tests, and two samples (F105 placenta and MB nasal) were positive in sgLAMP but negative in all other tests. We also observed two samples (M1 clitoral and M3 cervix swabs, Additional file 8) yielding ambiguous results (Additional file 8). As stated above, ‘ambiguous’ samples resulting in an orange colour post-amplification in cLAMP were reported as negative. The M3 cervix sample however, was determined to be positive (with clear yellow colour change) in *C. psittaci* cLAMP testing (Fig. 2).

**Table 5:** Comparison of EHV-1 LAMP assays utilizing rapidly processed swabs to qPCR testing

**EHV-1 rtLAMP**	**EHV-1 qPCR**			
		Positive	Negative	Total
	Positive	2	0	2
	Negative	2	30	32
	Total	4	30	34
	Kappa (0.95 CI; p(Kappa))	0.638 (0.185–1.09; 0.000)		
	McNemar’s Chi square (p(Chi sq))	0.500 (0.479)		
	Overall agreement ^b^	94.12% (66.67% PA; 96.77% NA)		
**EHV-1 cLAMP**^***#***^	**EHV-1 qPCR**			
		Positive	Negative	Total
	Positive	2	1	3
	Negative	2	27	29
	Total	4	28	32
	Kappa (0.95 CI; p(Kappa))	0.520 (0.047–0.993; 0.0014)		
	McNemar’s Chi square (p(Chi sq))	0.00 (1.00)		
	Overall agreement ^b^	90.62% (57.14% PA; 94.47% NA)		
**EHV-1 sgLAMP**	**EHV-1 qPCR**			
		Positive	Negative	Total
	Positive	2	2	4
	Negative	2	28	30
	Total	4	30	34
	Kappa (0.95 CI; p(Kappa))	0.433 (−0.029–0.896; 0.006)		
	McNemar’s Chi square (p(Chi sq))	0.250 (0.617)		
	Overall agreement ^b^	88.24% (50.00% PA; 93.33% NA)		

^#:^ two samples were excluded from analyses as immediate discoloration of the mix (to yellow and/or orange) was observed upon addition the swab suspension; ^b^: positive agreement (PA) and negative agreement (NA) are outlined in brackets

### LAMP in clinical setting

In order to assess the LAMP technology for POC deployment, we performed pilot testing of clinical samples using the *C. psittaci* LAMP assays on two separate occasions at Scone Equine Hospital Laboratory, New South Wales, Australia. Technicians with no prior molecular experience easily performed the assay after short (up to three days) training. Training included: sample processing, primer and reaction preparation, use of Genie Fluorometer for rtLAMP, and interpretation of results. On both occasions, we noted time from rapid sample processing to interpretation of results when assays were performed by technicians with and with no prior molecular diagnostic experience. On average, a technician with prior experience took 1 h to rapidly process and test up to eight samples using the rt- and/or cLAMP assays, while for newly trained technicians took 1.5 h. The assays were demonstrated to be robust, and easy to perform at our POC setting.

### 2017 And 2019 pilot testing

In 2017, a total of 34 fresh and frozen foetal tissue and placental swab samples from equine abortion events were tested with *C. psittaci* rtLAMP (Additional file 9), yielding 100% agreement with qPCR assay results (Table 6).

**Table 6:** Comparison of the POC *C. psittaci* LAMP results with the qPCR results

**2017 Testing**				
***C. psittaci*rtLAMP**	***C. psittaci*qPCR**			
		Positive	Negative	Total
	Positive	16	0	16
	Negative	0	18	18
	Total	16	18	34
	Kappa (0.95 CI; p(Kappa))	1.000 (1.000–1.000; 0.000)		
	Overall agreement	100%		

In 2019, a total of the 11 (eight retrospective frozen and three prospective fresh) foetal tissue and placental swab samples from equine abortion events were opportunistically tested for *C. psittaci* using cLAMP assay (Additional file 9). Note, we chose to test the cLAMP assay opposed to the sgLAMP assay to avoid additional steps (e.g., adding SYBR Green dye post incubation) which may further decrease utility of POC assays in a clinical setting [21]. As observed above, c- and sgLAMP are of comparable sensitivity and specificity. Prior to DNA extraction from the swab suspensions, we also performed rtLAMP testing to evaluate agreement with the POC cLAMP testing. The POC cLAMP and rtLAMP performed in the laboratory were in 100% agreement (Additional file 9). A discrepant result was noted for sample Fresh_FoalA_Nasal which tested negative by cLAMP, yielded amplification time in rtLAMP, however was considered negative as this was below the detection limit. This sample was positive using in house qPCR with an average Ct value of 30.07, indicating a low infectious load of 28 copies/μl of DNA based on the standard curve. However, as this was done using 11 samples only, we cannot accurately assess agreement between cLAMP and qPCRs.

## Discussion

Although attractive to use and available for many veterinary and human pathogens, LAMP technology is still in the early stages of POC and/or field use [13, 22–24]. This study describes reliable POC options for the detection of the equine pathogens: *C. psittaci* and EHV-1. Furthermore, our testing of ‘real-world’ clinical samples (e.g. equine mucosal swabs) in equine clinical setting represent a proof-of-concept that POC isothermal diagnostics can be applied to rapid disease screening in the equine industry.

During the initial LAMP assay optimisation, previously published [14, 19] and newly designed primer sets were confirmed to be species specific, and most importantly, as sensitive as gold-standard qPCR assays. LAMP assays run in the Genie III fluorometer were straightforward in the interpretation of results, with positive results being easily characterizable by amplification time and specific melt curves. While we described use of a real-time fluorometer for performing *C. psittaci* rtLAMP assays in our previous study [14], there are limited studies describing EHV-1 LAMP assays using this portable instrument. Recent study also used Genie III fluorometer to perform EHV-1 LAMP assays that can differentiate between neuropathogenic and abortigenic EHV-1 genotypes, however this LAMP assay also includes fluorophore labelled quencher probe and fluorescent primer in addition to LAMP primers [20]. In previous studies, the end point results for these assays were interpreted as visible bands on an agarose gel after electrophoresis and by visual confirmation of colour change from orange to green after addition of calcein for EHV-1 [16, 19].

To the best of our knowledge, both DNA targets were evaluated here for the first time with commercial colorimetric and SYBR Green dyes as end-point assays. The colorimetric LAMP assays, with 10 copies/μL determined as 100% detection limit, were slightly less sensitive than the rtLAMPs. Interestingly, the commercial colorimetric mix also gave false positive results for EHV-1 when using the primer set described by Nemoto et al. [19], whereas EHV-1 primers designed in this study did not cause any false positive test results. Other studies using this colorimetric method also observed false positive test results, which may have arisen due to primer dimerization, non-specific amplification due to primer design, change of pH or other factors [13, 25, 26]. Moreover, we demonstrated that the LAMP reagents were thermostable at a storage temperature of 4 °C for at least six weeks**.**

In order to validate the rtLAMP assays from this study, two accredited Veterinary Diagnostic laboratories performed comparative reference qPCRs on 88 *C. psittaci*, and a total of 43 EHV-1 DNA samples (35 tested by EMAI, and eight by IDEXX). *C. psittaci* rtLAMP was in 98.86% agreement with the reference qPCR assay, confirming its sensitivity and specificity of the assay and its suitability for POC diagnostic use. Although preliminary validation of 43 samples indicated 91.43 and 100% agreement for EHV-1 rtLAMP respectively, with the reference qPCR assays run at two different laboratories, additional comparison testing with a larger sample set is required.

As a proof of concept for POC use, we previously evaluated a rapid swab processing technique employing a simple vortexing and heat lysis which allowed the application of crude DNA extracts in isothermal detection of *C. pecorum*, a livestock and koala pathogen [14]. As demonstrated by Akter and colleagues, the use of mucosal and/or foetal tissue swabs (rather than directly testing foetal tissues) facilitates high throughput testing and minimises the presence of PCR inhibitors in found tissue extracts [27]. As LAMP has been shown to be tolerant to PCR inhibitors, we sought to evaluate the use of rapidly processed clinical swabs (foetal, nasal, placental, cervical, clitoral, vaginal, and penile) in our LAMP assays to a) allow for simple DNA extraction methods, and b) decrease the sample preparation time in the clinical setting. Overall, the test results from rapidly processed swab suspensions were highly congruent between the assays, and most importantly the in-house reference qPCR assays. However, swab samples with low infectious loads, such as the foal nasal swab from our study tested at the POC, could be missed in isothermal testing as positives using rapid swab processing method. In comparison, the in-house qPCR assay involved time-consuming DNA extraction using commercial kit, taking an estimated 2 h for 12 samples, and involving the use of a real-time qPCR instrument, quoted at USD $ 20,000. The swab suspension rtLAMP assays performed in the Genie III instrument were most congruent to the in-house qPCR DNA testing. However, the discrepant results observed were for swab samples with low loads on the border of LAMP lower detection limits.

No such trend was observed for discrepant results from the colorimetric LAMP assays (e.g., positive results by WarmStart and/or SYBR Green colorimetric assays was sometimes observed when the remaining three assays yielded negative results). These discrepant colorimetric results could be due to several reasons, such as pH change of the WarmStart Mix, DNA contamination from opening the tubes to add SYBR Green DNA, or non-specific amplification and/or primer dimerization [13, 17]. Nevertheless, colorimetric options such as the 3-step (i.e. prepare the mix and add template, incubate, visualize results) WarmStart® LAMP assays are also promising POC equine pathogen diagnostic tools that do not require expensive equipment and additional steps such as addition of DNA binding dyes post incubation [21].

In this study, to the best of our knowledge, we applied and evaluated the use of *C. psittaci* isothermal assays (albeit for limited number of samples) for the first time in an equine clinical setting in Australia. Rapid diagnosis of infectious causes of equine pregnancy loss is important for equine veterinarians and stud owners to determine appropriate disease treatment and management. In the case of *C. psittaci*, the information is also critical to alert attending staff to the zoonotic risk [1, 4, 14]. The isothermal assays were estimated to cost USD$ 4/reaction, which is inclusive of reagents (isothermal mix and primers) and general consumables (PCR tubes, filter tips). Additionally, a “one-off” setup cost may be incurred for the purchase of Genie III real-time fluorometer (Optigene, UK) if rtLAMP is to be run (quoted at USD$ 12,000); as opposed to a comparatively cheap heating block (quoted at up to USD$ 700). Further, vortexes and mini centrifuges are useful, but not necessary. The swab can be vigorously twirled to release the cells, whereas handheld, commercially available “fidget-spinner” can be used as a centrifuge platform [28].

On the 2017 occasion where we tested 34 swab samples, the *C. psittaci* rtLAMP assay, performed in Genie III, was demonstrated to be robust and easy to use. In 2019, we performed a much smaller pilot trial using cLAMP, performed on heating block with result denoted as a color change, on only 11 samples. The feedback about the POC use of these assays was that after training they were easy to use and interpret. On both occasions, we achieved 90–100% congruence between the LAMP assays and the reference qPCR, however we must note that we have tested *C. psittaci*-suspected abortion material that typically contains high pathogen loads and the colorimetric LAMP assay was only evaluated on 11 samples. A previous study showed that the *C. psittaci* infectious load (ranging from 1 × 10^2^ – 2 × 10^6^ genome copies/μl) is high in DNA extracted from placental and foetal tissues [4], thereby making the detection of the organism easy in these tissue swabs. Due to such limited numbers, and use of somewhat biased samples, further larger trials testing both high and low load samples are now warranted for *C. psittaci* and EHV-1 isothermal POC testing.

## Conclusion

If the LAMP assays are demonstrated to be reliable for a specific pathogen detection using ‘real world’ clinical animal samples then translation of ‘from lab to the stable’ diagnostic testing would be feasible [21, 22, 29]. Rapid and accurate POC diagnostics will benefit veterinarians by supporting timely (same day) diagnosis and advice to affected stakeholders to allow for timely treatment of affected horses and earlier interventions to protect unaffected horses on the same stud. In this study, we have demonstrated applicability of all three LAMP assays (real-time, colorimetric and SYBR Green) for the detection of two equine pathogens using rapidly processed clinical samples confirming POC suitability of these tests. The achievement of accurate results in minutes rather than hours presents a significant step forward in providing guidance for treatment and biosecurity decisions. Going forward, it is recommended that we apply further testing on a range of samples, assess additional sample preparation methods such as use of one-step DNA extraction and/or cell lysing buffers and the use of lyophilized reagents, and evaluate the use of the equine LAMP assays on stud farms.

## Methods

### Published and newly designed LAMP assays

For the detection of *C. psittaci*, we applied our previously described isothermal assay and primers targeting a 263 bp of the *C. psittaci*-specific ORF_607 gene [14]. For EHV-1 detection, previous isothermal assays targeted 209 bp of the EHV-1 glycoprotein E (gE) gene [16, 19]. Both targets are conserved among the strains, as evaluated in previous studies and additional blast analyses in our study using blastn for sequence specificity (https://blast.ncbi.nlm.nih.gov/Blast.cgi). In this study, we utilized both LAMP Designer 1.15 software which yields a single set of six LAMP primers (Premier Biosoft, CA, USA) and open-source Primer Explorer v5 software which yields five sets of four LAMP primers (Fujitsu Limited, Japan) to evaluate primer design for EHV-1 gene target, and to compare them to the previously published primers [16]. All newly designed primer sets were evaluated in silico using blastn for sequence specificity (https://blast.ncbi.nlm.nih.gov/Blast.cgi) and primer characteristics using online tools OligoEvaluator (http://www.oligoevaluator.com) and OligoAnalyzer (https://sg.idtdna.com/pages/tools/oligoanalyzer). Following sequence analyses and preliminary isothermal testing with the sets of newly designed primers (as to evaluate time to amplify, amplification and melt curves for each set), we selected the most optimal primer set for EHV-1, designed by Primer Explorer. As the Primer Explorer yields set of four LAMP primers (F3, B3, FIP and BIP), we additionally designed LF and LB primers (Additional files 1, 2, and 3).

### Newly designed EHV-1 and *C. psittaci* real-time LAMP (rtLAMP) assays evaluation

The newly designed and previous primer sets for EHV-1 were tested and compared in rtLAMP assay using quantified DNA, extracted from viral culture. Similarly, the *C. psittaci* primer set was again evaluated in this study (Additional file 3). All rtLAMP assays were performed in 25 μL reaction volumes, consisting of 15 μL Isothermal Master Mix ISO001 (Optigene, UK), 5 μL primers mix (at 0.2 μM F3 and B3, 0.8 μM FIP and BIP, and 0.4 μM LF and LB), and 1 μL (for sensitivity and LOD assays only where we used quantified DNA) or 5 μL template (for testing clinical samples, as recommended by the manufacturers); run at 65 °C for 30 min followed by denaturation step of 98–80 °C at a rate of 0.05 °C/s in the Genie III real-time fluorometer (Optigene, UK) to create a high-resolution melt curve (Fig. 1A and B). A positive (targeted organism DNA) and negative (MilliQ water) controls were included in each assay. For each species-specific rtLAMP assay, the limit of detection was evaluated using 1 μL of quantified DNA in serial dilutions from 10^2^ (tested in triplicate) to 10^− 1^ copies/μl (tested as five replicates) (Additional file 4). Each serial dilution set (10^2^ to 10^− 1^ copies/μl) was prepared separately for each replicate, from the same quantified DNA (from *C. psittaci* Horse_Pl, and EHV-1 V#2019–02 strains, respectively). Dilutions were tested in separate runs by three operators.

### Target specificity of rtLAMP assays

The specificity of *C. psittaci*, and newly developed EHV-1 rtLAMP assays was evaluated against a range of previously screened and confirmed DNA extracts from other viruses, cultured bacteria and horse tissues (Additional file 4).

### Colorimetric LAMP assays with end-point “naked eye” interpretation of results

#### Commercial colorimetric end-point LAMP (cLAMP) assays

Colorimetric LAMP (cLAMP) assay using 2X WarmStart® Colorimetric LAMP Master Mix (New England BioLabs, USA) was performed in 25 μL reaction volumes, consisting of 12.5 μL 2X WarmStart® Colorimetric LAMP Master Mix, 2.5 μL primers mix (at 0.2 μM F3 and B3, 1.6 μM FIP and BIP, and 0.4 μM LF and LB as per manufacturer recommendation) and 1 μL (for sensitivity assays) or 5 μL (for clinical samples) template, run at 65 °C for 30 min on a thermal block. Using the colorimetric method, reactions must be bright pink in colour prior to amplification, indicating that the reaction has a high pH, which is essential for the pH-dependent colorimetric LAMP reaction. Furthermore, the primers and DNA template should be used as an aqueous solution (with a neutral pH in the range of 7–7.4) so as not to interfere with the required pH for the colorimetric LAMP assay. Post amplification, the tubes were cooled to room temperature and examined by eye. Positive (target species DNA) and negative (MilliQ water and/or LAMP mix only) controls were included in each assay. As per manufacturers chart, positive reactions turned yellow, while negative reactions remained pink (Fig. 1C). If a colour change was not clearly evident, (e.g. an orange colour was visible), the reactions were incubated at 65 °C for an additional 5–10 min as per manufacturer’s instructions. If the colour change was still not distinct after additional incubation, the result was deemed “ambiguous” and was considered negative. For each species-specific colorimetric LAMP assay, limit of detection was evaluated using 1 μL of quantified DNA for serial dilutions from 10^2^ (tested in triplicate) to 10^− 1^ copies/μl (tested in five replicates) (Additional file 4).

#### SYBR green LAMP assays (sgLAMP)

10,000X Sybr Green I dye (Sigma Aldrich, Australia) was diluted to 1000x in PCR-grade water in a total of volume of 1 ml. The sgLAMP assays were performed in 25 μL reaction volumes, consisting of 15 μL No Dye Isothermal Master Mix ISO001 (Optigene, UK), 5 μL primers mix (at 0.2 μM F3 and B3, 0.8 μM FIP and BIP, and 0.4 μM LF and LB) and 1 μL (for sensitivity assays) or 5 μL template (for testing samples), run at 65 °C for 30 min on a thermal block. After 30 min incubation, 2 μl of 1000x SYBR Green dye was added to the tubes. A visible colour change from orange (indicating no amplified product, negative sample) to bright yellowish green (indicating amplified product, positive sample) was used for results interpretation (Fig. 1D). Positive (targeted species DNA) and a negative (MilliQ water and/or mix only) controls were included in each assay. For each species-specific sgLAMP assay using the previous *C. psittaci* and newly developed EHV-1 primers, limit of detection was evaluated using 1 μL of quantified DNA in serial dilutions from 10^2^ (tested in triplicate) to 10^− 1^ copies/μl (tested in five replicates) (Additional file 4).

### Stability of the LAMP reagents

In order to test the robustness of our LAMP assays and stability of the reagents, the three (rt, c, sg) LAMP isothermal master mixes and primers were kept refrigerated (4 °C) for up to six weeks. To test the stability of the reagents, serially diluted, purified and quantified *C. psittaci* gDNA (10^5^, 10^3^ and 10^2^ genome copies/μl) was tested weekly for a period of six weeks. The isothermal master mixes were assayed as outlined above every week using 1 μl of the serial-diluted *C. psittaci* DNA (Additional file 5) as a template.

### Validation of the rtLAMP assays

The *C. psittaci*, and the newly developed EHV-1 rtLAMP assays were evaluated against DNA samples, extracted from swabs from equine abortion cases, that had been previously tested with: a) the reference *C. psittaci* [30] (*n* = 88 samples) and EHV-1 [31] (*n* = 35 samples) diagnostic qPCR assay at Elizabeth Macarthur Agricultural Institute (EMAI) Veterinary Diagnostic Laboratory, Menangle, NSW, Australia; or b) reference singleplex EHV-1 RealPCR™ (*n* = 8 samples) diagnostic qPCR assay at IDEXX Veterinary Diagnostic Laboratory, Brisbane, QLD, Australia (Additional file 7). These are National Association of Testing Authorities, Australia (NATA) accredited (ISO17025) laboratories.

### Rapid sample processing

A method for rapid specimen processing was also evaluated on a total of 36 horse mucosal (foetal, nasal, placental, cervical, clitoral, vaginal, and penile) swabs for use with the LAMP assays in order to reduce the time involved in sample processing and DNA extraction prior to the diagnostic assay. All the swabs were collected by qualified veterinarians as a part of routine health and diagnostic investigations. The testing and use of these swabs were approved by University of the Sunshine Coast Animal Ethics approval exemption (ANE1719, ANA19149; ANE1939).

Of the 36 collected swabs, 24 were a regular wood shaft cotton tip dry swab in individual tubes (Copan, Italy), and the remaining 12 were individual Transystem plastic shaft Amies gel swabs (Copan, Italy). The rapid swab processing was performed in a Biosafety Cabinet. A 300 μl aliquot of sterile MilliQ water was added to sterile 1.5 ml Eppendorf tubes. The dry and/or Amies gel swabs were placed in tubes and the shaft was cut off so that the swab fit within the tube. For Amies gel swabs, if present, any excess gel media was carefully removed. The mucosal swabs were vortexed for 1 min and heat lysed at 95 °C for up to 10 min. The swab suspension was briefly centrifuged, cooled to room temperature and a 5 μl aliquot was then added to the LAMP reactions as outlined above (Additional file 6). Based on limit of detection experiments outlined above, in rtLAMP a swab suspension sample was considered negative for both *C. psittaci* and EHV-1 if amplification time was > 23.50 min as this was considered below detection level (BDL). In the c- and sgLAMPs a sample was considered negative if there was no clear colour change (i.e. colour change was ambiguous). Inhibition of amplification by swab suspensions was also assessed with ‘spiking’ experiments, where we added 10 μl of *C. psittaci* and/or EHV-1 DNA to five 25 μl aliquots of each swab suspension that tested negative. In order to perform comparison to the reference *in-house* qPCR assays, the remaining volume of the swab suspension was used for DNA extraction using QiaAMP DNA mini kit, as per manufacturer instructions (Qiagen, Australia) (Additional file 3).

### Comparative in-house qPCR assay

The in-house qPCR assays for *C. psittaci* and EHV-1 targeted amplicon generated by previously described F3 and B3 primers [14, 19]. Briefly, the *C. psittaci* qPCR assay targeted a 263 bp fragment of the Cps_ORF607 gene characterised with a melt of 78 ± 0.5 °C [14, 32], while the EHV-1 qPCR assay targeted a 209 bp of the *g*E gene characterised with a melt of 82.5 ± 0.5 °C [19]. All qPCR assays were carried out in a 15 μL total volume, consisting of 7.5 μL iTaq Master Mix (Biorad, Australia), 0.5 μL of each 10 μM forward and reverse primer (Sigma Aldrich, Australia), 3.5 μL MiliQ water, and 3 μL DNA template. The qPCR assays were run for 40 cycles, and in each qPCR assay a positive (target organism DNA) and negative (mix only and MiliQ water) controls were included. Each sample was tested in duplicate. The assays were calibrated using known standards of *C. psittaci* Horse_Pl, and EHV-1 V#2019–02 strains (Additional file 4) DNA serially diluted from 10^6^ to 10^0^ copies/μl. Samples with a cycle threshold (Ct) > 35 and below the signature melt threshold were considered negative (Additional file 8). Both LAMP and qPCR testing were performed in a semi-blind fashion, by three different operators. All samples were screened for the presence of equine DNA, targeting 168 bp of the equine *Cyt*B gene [33].

### LAMP assays at the POC

In order to assess the use of these LAMP assays at the POC or in the clinical setting, testing was performed at Scone Equine Hospital (SEH) Laboratory, NSW, Australia, on two separate occasions. In August 2017, 34 rapidly processed horse mucosal and tissue swabs were tested with the *C. psittaci* rtLAMP assay. In July 2019, the *C. psittaci* cLAMP assay was opportunistically applied on: eight retrospective frozen foetal and placenta swab samples collected from previous 2016/2017 foaling seasons; and three prospective placental and foetal dry swab samples (Additional file 9). On both occasions, we utilized two separate areas in the laboratory: “sample preparation” and “PCR clean” area. General laboratory equipment (pipettes, vortex, mini centrifuge, heating block) was utilised, and technicians with no prior molecular diagnostic experience as well as those with prior molecular diagnostic experience were running the assays. All swab samples were rapidly processed in a “sample area” in a biosafety cabinet by vortexing (to detach cells from the swab) followed by heat lysis at 90 °C for 5–10 min to release DNA as outlined above. In a separate “clean area”, isothermal mix was prepared and aliquoted in PCR tubes. Negative controls containing MilliQ water and/or mix only were set aside, while the closed sample tubes were brought back to the clean bench in the “sample area” for swab suspension addition.

On both occasions, after POC testing the same swab suspension samples were sent to the University of the Sunshine Coast Research Laboratory for confirmatory diagnostic testing using *C. psittaci* qPCR assay (all samples from 2017 and 2019 visit) and rtLAMP assay (samples from 2019 visit) (Additional file 9).

### Statistical analyses

We compared the results for: a) the same DNA samples previously tested with reference assays run in the veterinary diagnostic laboratories, with the rtLAMP assays; b) rapidly processed swabs suspensions tested with the real-time and colorimetric LAMP assays; c) rapidly processed swabs tested with LAMP and with DNA extracs of the same samples tested with in-house qPCR assays; and, d) samples tested at the POC using rapid processing and LAMP and DNA extracts from the same samples with in-house qPCR assays. The performance of the tests evaluated on the same samples was done using EpiTools online epidemiological calculators using diagnostic test evaluation modules (https://epitools.ausvet.io/comparetwotests) [34]. For DNA samples tested in diagnostic laboratories and in this study by rtLAMP, we used test evaluation against a gold standard module, which included sensitivity, specificity and positive and negative likelihood ratios, with results presented as estimates of sensitivity and specificity with specified Clopper-Pearson (exact) confidence limits and point estimates of positive and negative likelihood ratios, and overall test congruence. For swab suspension samples tested by LAMPs and in house qPCRs, we compared the performance of two tests evaluated on the same unknown/mixed status samples with calculated Kappa and positive and negative agreement proportions, as well as McNemar’s Chi-squared value with 95% confidence. It is suggested the Kappa value be interpreted as follows: values ≤0 as indicating no agreement and 0.01–0.20 as none to slight, 0.21–0.40 as fair, 0.41–0.60 as moderate, 0.61–0.80 as substantial, and 0.81–1.00 as almost perfect agreement.

## Supplementary Information

**Additional file 1.** Newly designed EHV-1 LAMP primers sequences utilized in this study. Primer sequences and their lengths are outlined in the table.

**Additional file 2.** EHV-1 LAMP primer sequences and positions in the target gene regions. In this figure, we outline the EHV-1 gene target, where previously described primers are indicated in orange, while the newly designed primers are indicated in purple.

**Additional file 3.** Workflow for development, evaluation and application at the POC for the equine isothermal assays. In this diagram we present the utilised workflow.

**Additional file 4.** Isothermal assays sensitivity and specificity comparisons. In this table, time to amplify (min:ss) and HRM (oC) melts, and colour change results are presented for serially diluted C. psittaci (sheet 1) and EHV-1 (sheet 2) DNA to evaluate detection limits of isothermal assays. On sheet 3, we denote results for rtLAMP amplification using a range of bacterial and viral DNA.

**Additional file 5.** Stability and reproducibility of the isothermal assays with components kept on 4 °C. In this table, we present results for real-time and colorimetric isothermal amplification using same DNA templates over 6-week period when reagents were kept on 4 °C.

**Additional file 6.** Rapid swab processing. A: Clinical swabs available for testing, from left: placental and foetal swabs; Mare nasal, vaginal and cervical swabs; B: Vortexed swabs in 300 μl sterile MilliQ water; C: Heat lysis of the swab suspension; D: Heat lysed swab suspension used as a template in isothermal testing. The images were taken with iPhone SE. In this image, we present step-by-step rapid swab processing method.

**Additional file 7.** Validation of LAMP testing (reported as time to amplify (min:ss), and signature melt in (°C) using diagnostic laboratory tested samples. In this table, we present (paired) results of reference diagnostic assay and our isothermal assays when testing the same samples.

**Additional file 8.** Clinical samples testing. In this table, we present (paired) results of our three isothermal assays compared to reference qPCR assays when testing the same samples. On sheet 1 are results for C. psittaci, and on sheet 2 for EHV-1 detection, respectively.

**Additional file 9.** Testing at the POC. In this table, we present (paired) results of C. psittaci isothermal assays, performed at the POC in Scone Equine Hospital on two separate occasions, compared to reference qPCR assay.

## Data Availability

Data, such as the primer sequences, Genie III outputs (time to amplify in min and sec, and HRM in ^o^C), the colour change and end-point interpretations and qPCR outputs (average Ct values and HRM in ^o^C) generated or analysed during this study are included in this published article [and its supplementary information files]. The data used and/or analysed (such as the Genie III and qPCR runs outputs, and images for colour changes) during the current study are available from the corresponding author on reasonable request.

## Abbreviations

bp: base pairs
Ct: Cycle threshold
*C. psittaci*: *C. psittaci*
EHV-1: Equine Herpes Virus 1
LAMP: Loop-mediated isothermal amplification
cLAMP: colorimetric LAMP
rtLAMP: real-time LAMP
sgLAMP: SYBR Green LAMP
PCR: polymerase chain reaction
POC: Point of Care
qPCR: Quantitative polymerase chain reaction
