# Supplementary material for: Real-time fluorometric and end-point colorimetric isothermal assays for detection of equine pathogens C. psittaci and equine herpes virus 1: validation, comparison and application at the point of care
Source: BMC Vet Res. 2021 Aug 19;17:279. doi: 10.1186/s12917-021-02986-8 (PMC8375077; doi:10.1186/s12917-021-02986-8)
Supplement: Supplementary file 1 — Additional file 1. Newly designed EHV-1 LAMP primers sequences utilized in this study. Primer sequences and their lengths are outlined in the table. [file 12917_2021_2986_MOESM1_ESM.docx]

Additional File 1. Newly designed EHV-1 LAMP primers sequences utilized in this study.

Primer sequences and their lengths are outlined in the table.

| **Primer name** | **Sequence 5’ to 3’** | **Primer length (bp)** | **F3/B3 amplicon**  **length (bp)** |
| --- | --- | --- | --- |
| **EHV-1 gE gene** | | | |
| F3 | ACGTTTCCATCGACGGAATG | 20 | 216 |
| B3 | ACTCCAGGTTCACCGACAG | 19 |  |
| FIP (F1c-F2) | CGGGGTCTTAACGCGCAAGCGTGCTGCTCACCGTCAAG | 38 |  |
| BIP (B1c-B2) | CCGTTCCTCAGGTTCCCGTAAAGATTCGCCATCAGCGTACA | 41 |  |
| LF | TTGCGGTTGTTTGGGCG | 17 |  |
| LB | CACGGATTTTGTGGTGCACG | 20 |  |
