# Supplementary figures and images for: Real-time fluorometric and end-point colorimetric isothermal assays for detection of equine pathogens C. psittaci and equine herpes virus 1: validation, comparison and application at the point of care
Source: BMC Vet Res. 2021 Aug 19;17:279. doi: 10.1186/s12917-021-02986-8 (PMC8375077; doi:10.1186/s12917-021-02986-8)

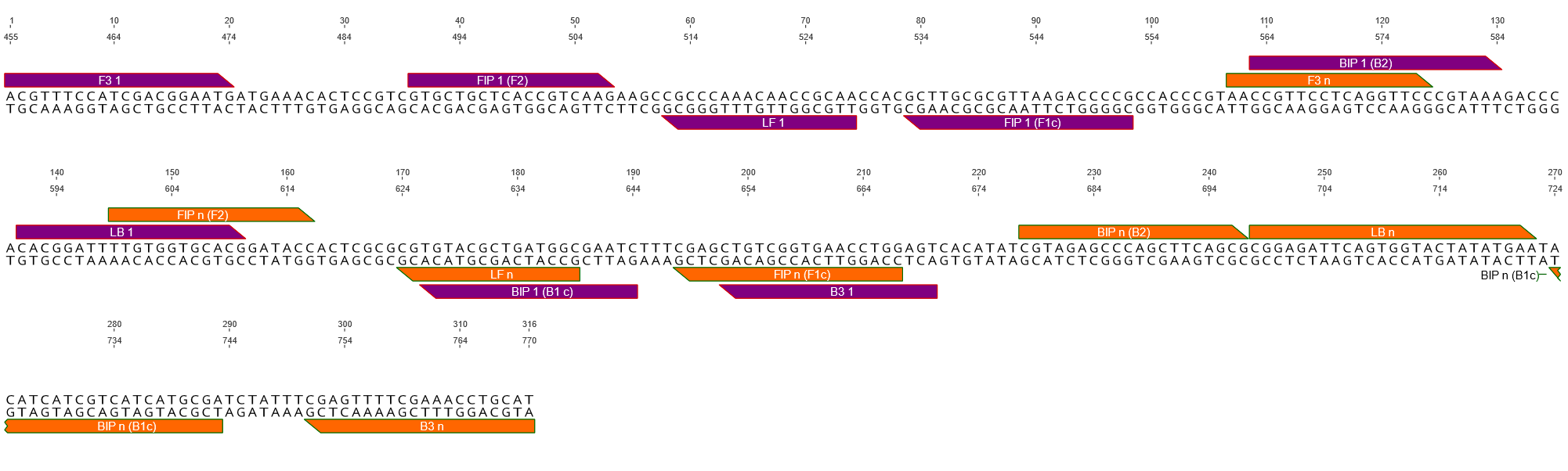

Supplement: Supplementary file 2 — Additional file 2. EHV-1 LAMP primer sequences and positions in the target gene regions. In this figure, we outline the EHV-1 gene target, where previously described primers are indicated in orange, while the newly designed primers are indicated in purple. [file 12917_2021_2986_MOESM2_ESM.png]

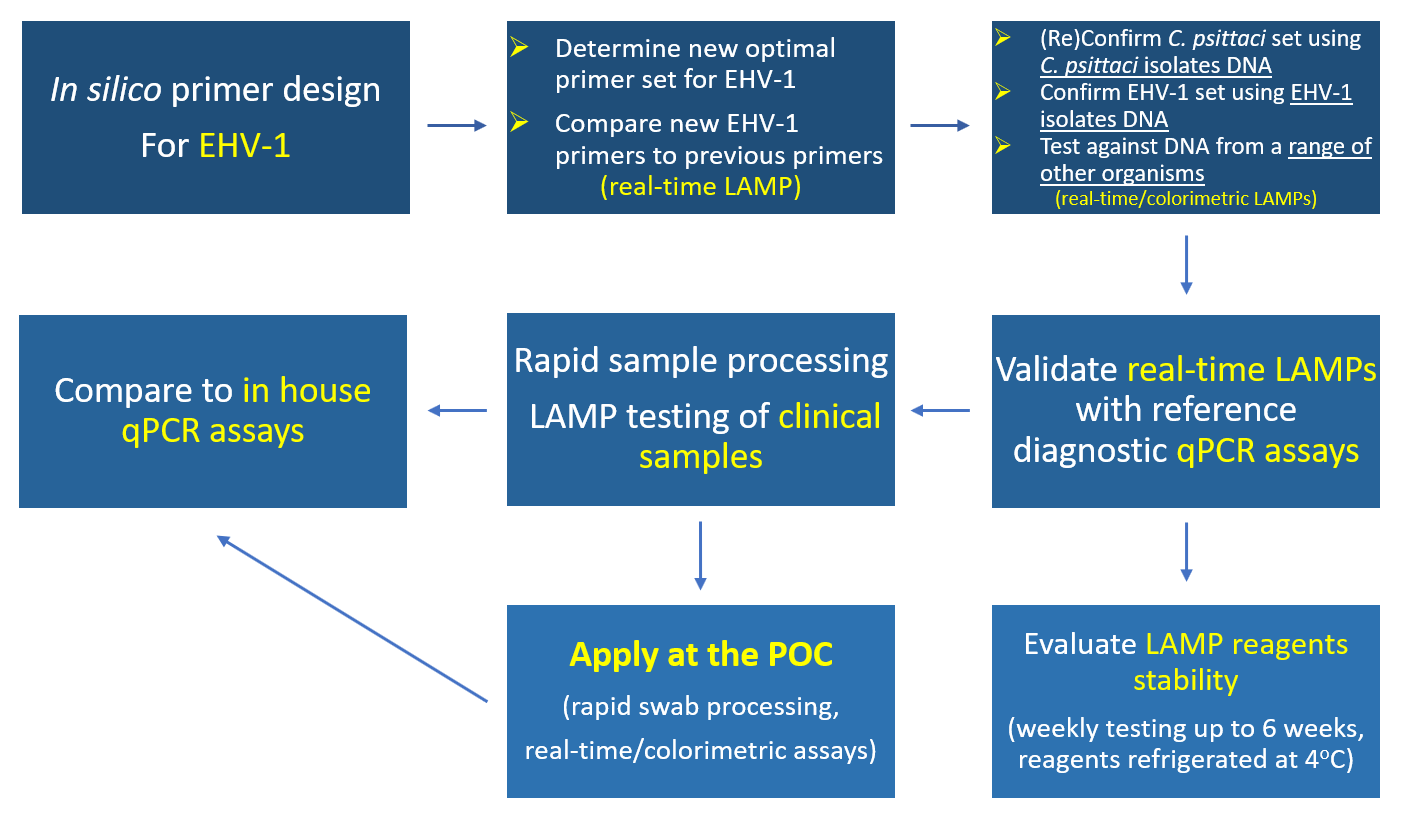

Supplement: Supplementary file 3 — Additional file 3. Workflow for development, evaluation and application at the POC for the equine isothermal assays. In this diagram we present the utilised workflow. [file 12917_2021_2986_MOESM3_ESM.png]

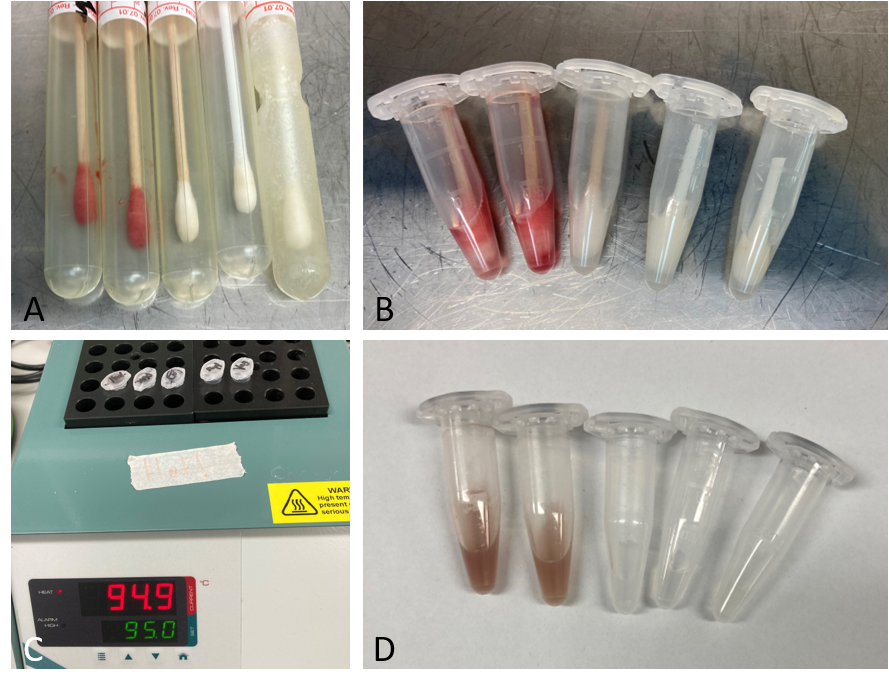

Supplement: Supplementary file 6 — Additional file 6. Rapid swab processing. A: Clinical swabs available for testing, from left: placental and foetal swabs; Mare nasal, vaginal and cervical swabs; B: Vortexed swabs in 300 μl sterile MilliQ water; C: Heat lysis of the swab suspension; D: Heat lysed swab suspension used as a template in isothermal testing. The images were taken with iPhone SE. In this image, we present step-by-step rapid swab processing method. [file 12917_2021_2986_MOESM6_ESM.png]
